# Synergistic Effect of Exogenous Application of Proline and Boric Acid on the Growth, Physiological Aspects, and Postharvest Quality of Radish under Salt Stress

**DOI:** 10.1021/acsomega.5c03010

**Published:** 2025-07-21

**Authors:** John Victor Lucas Lima, Larissa Nicácio Pessoa, Maria de Fátima Duarte de Souza Neta, Jhon Wadner Kerson Phanord, Pablo Henrique de Almeida Oliveira, Antonio Gideilson Correia da Silva, Ayslan do Nascimento Fernandes, Fagner Nogueira Ferreira, Ester dos Santos Coêlho, Aurélio Paes Barros Júnior, João Everthon da Silva Ribeiro

**Affiliations:** Department of Agronomic and Forestry Sciences, 74384 Universidade Federal Rural do Semi-Árido, Mossoró, Rio Grande do Norte 59625-900, Brazil

## Abstract

Salinity is one of the primary abiotic stresses that limits yield, particularly in semiarid regions, affecting the growth and physiological aspects of crops. Proline and boric acid, when applied exogenously, can improve plants’ tolerance to salt stress. Therefore, this study aimed to evaluate the effect of exogenous proline and boric acid application on the growth, physiological aspects, and postharvest quality of the “Crimson Gigante” radish variety under salt stress. The research was conducted in a greenhouse using a randomized block design with a factorial scheme of 3 × 4 and four replications. The treatments were composed of three levels of electrical conductivity of the irrigation water (0.5 dS m^–1^, 2.5 dS m^–1^, and 4.5 dS m^–1^) and four treatments with attenuating agents [control, proline (5 mM), boric acid (1 mM), and the combination of proline and boric acid (5 mM + 1 mM)]. Growth variables, gas exchange, photosynthetic pigments, and postharvest physicochemical quality were evaluated. Applying proline and boric acid improved plant growth and physiological responses under salt stress (2.5 and 4.5 dS m^–1^). At 2.5 dS m^–1^, proline + boric acid increased the plant height (11.96%), stem diameter (29.40%), and photosynthetic rate (19.27%). At 4.5 dS m^–1^, the same combination enhanced plant height (25.69%), shoot dry mass (36.70%), and pulp firmness (30.78%). Boric acid increased chlorophyll a (19.88%) and anthocyanins (26.91%) at 2.5 dS m^–1^. Proline raised flavonoids (45.05%) and anthocyanins (55.18%) at 4.5 dS m^–1^. Thus, the combined application of proline and boric acid (5 + 1 mM) may be a viable alternative to mitigate the deleterious effects of salt stress in radish plants.

## Graphical abstract

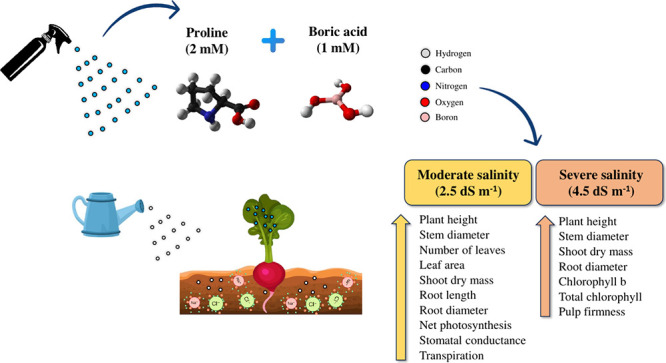

## 1 Introduction

Salinity of irrigation water is one of the main factors limiting agricultural production in several regions, especially in arid and semiarid climates.^1–3^ Salt stress negatively affects plant physiological processes, including water absorption, net CO_2_ assimilation, stomatal opening, and root growth, leading to decreased yield and postharvest quality of crops.^4–7^ In addition, the high salt content in irrigation water can also induce the accumulation of toxic substances, such as sodium and chlorine, in plant cells, causing cell damage and affecting plant defense mechanisms.^8,9^

Several strategies have been employed to mitigate the effects of salinity on plants, highlighting the use of exogenous substances that promote stress tolerance.^10–13^ Among the strategies, the exogenous application of compounds such as proline and boron has shown promising results in several crops.^14–17^ These compounds can enhance plant adaptation under salt stress conditions by modulating physiological mechanisms that involve osmotic regulation, cell membrane stability, and antioxidant activity.^18,19^

Proline, an amino acid that acts as an osmoprotectant, has been applied exogenously to improve plant tolerance to salt stress.^20,21^ This amino acid helps maintain cellular water balance, protects cells from damage caused by reactive oxygen species (ROS), and contributes to the stability of proteins and membranes.^22^ The exogenous application of proline has shown benefits in growth, gas exchange, and photosynthetic pigments of plants under salt stress.^23,24^

Boron, an essential micronutrient for plant growth, has shown potential in mitigating salt stress in crops such as cotton,^25^ soybean, wheat,^26^ and barley.^27^ Its exogenous application, usually in boric acid, maintains cell membrane integrity and modulates antioxidant responses, increasing fruit growth and quality under salinity conditions.^28,29^ Additionally, this nutrient is crucial for lignin synthesis, sugar transport, and cell wall development and is essential for plant growth and development.^30,31^

The combined use of proline and boric acid as attenuators of saline stress has not been well explored. Although studies have examined the isolated effects of each compound,^28,32^ little information exists on the combined use of these substances. The joint exogenous application (proline + boric acid) can strengthen cellular protection mechanisms, optimize physiological processes, and increase plant tolerance to saline stress.

Radish (*Raphanus sativus* L.), belonging to the Brassicaceae family, is widely cultivated due to its short growth cycle and low production cost.^33^ In addition to its economic importance, this vegetable is rich in nutrients, including vitamin C and a complex of B vitamins, as well as minerals such as potassium, calcium, and magnesium, which are beneficial for cardiovascular and digestive health.^34^ Radish is also used in traditional medicine in some cultures due to its antioxidant and anti-inflammatory properties.^35^ However, radishes are sensitive to salinity, which compromises their growth and quality.^36^ Radish was selected as a model crop due to its short growth cycle, nutritional value, and economic relevance.

Although both proline and boric acid have been studied individually as stress mitigators, their combined effect on radish under saline conditions has not been previously investigated. This novel approach seeks to determine whether their joint application can provide superior physiological and postharvest benefits compared with the use of proline or boric acid alone. Therefore, our hypotheses are that (1) the exogenous application of proline and boric acid improves the growth and physiological aspects of radish under salt stress, (2) the combination of these compounds results in a synergistic effect, promoting a better tolerance of plants to salt stress, and (3) the postharvest quality of radish roots will be improved with the joint application of these compounds. Given the above, this study aimed to evaluate the synergistic effect of exogenous proline and boric acid application on the growth, physiological aspects, and postharvest quality of radish under salt stress.

## 2 Results

Growth variables were influenced by the application of attenuators under different salinity levels (Figure 1A–D). At a moderate salinity level (2.5 dS m^–1^), the combined application of proline and boric acid promoted the largest increases in most variables, with an increase of 11.96% in plant height (Figure 1A), 29.40% in stem diameter (Figure 1B), 33.33% in leaf number (Figure 1C), and 36.86% in leaf area (Figure 1D). At a higher salinity level (4.5 dS m^–1^), the treatment that used the combined attenuators (proline + boric acid) continued to stand out for plant height, increasing by 25.69% (Figure 1A). However, for the stem diameter, the isolated application of proline presented the highest value (19.16%), followed by boric acid (14.56%), whereas the combined application of both attenuators led to a smaller increase (6.25%) (Figure 1B). Regarding the number of leaves at the saline level of 4.5 dS m^–1^, boric acid applied alone was more effective, with an increase of 19.23% (Figure 1C).

**Figure 1:**
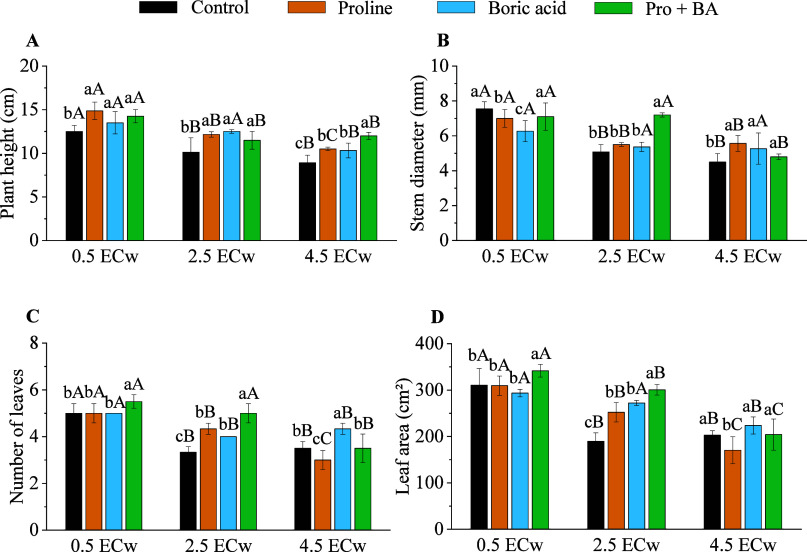
Plant height (A), stem diameter (B), number of leaves (C), and leaf area (D) of radish plants under levels of electrical conductivity of irrigation water (ECw) and exogenous application of proline, boric acid, and proline + boric acid (Pro + BA). Means followed by the same lowercase letters do not differ for attenuations, and means followed by the same uppercase letters do not differ for ECw by Tukey’s test at 5% probability.

Shoot dry mass (SDM) was influenced by attenuators under salt stress conditions (Figure 2A). At a level of 4.5 dS m^–1^, the application of proline resulted in an increase of 33.16% compared to the control, while boric acid promoted an increase of 30.40% (Figure 2A). The combination of proline and boric acid resulted in the largest increase in this saline level at 36.70% (Figure 2A). At 2.5 dS m^–1^, the proline and boric acid combination resulted in an increase by 12.22% compared to the control (Figure 2A). Root length (RL) also varied according to the attenuating factor (Figures 2B and[Fig fig3]). At 2.5 dS m^–1^, the application of boric acid resulted in an increase by 23.95%, while the combination of proline and boric acid raised this value to 26.60% (Figure 2B).

**Figure 2:**
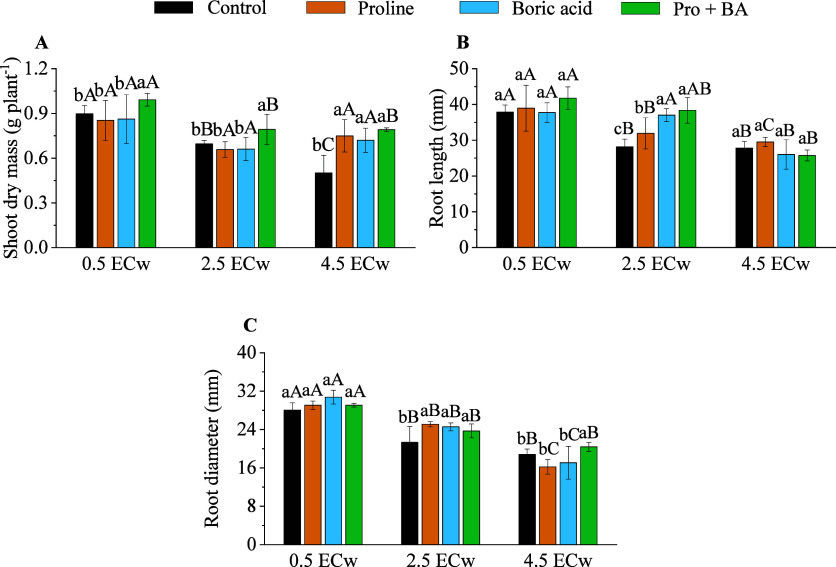
Shoot dry mass (A), root length (B), and root diameter (C) of radish plants under levels of electrical conductivity of irrigation water (ECw) and exogenous application of proline, boric acid, and proline + boric acid (Pro + BA). Means followed by the same lowercase letters do not differ for attenuations, and means followed by the same uppercase letters do not differ for ECw by Tukey’s test at 5% probability.

**Figure 3:**
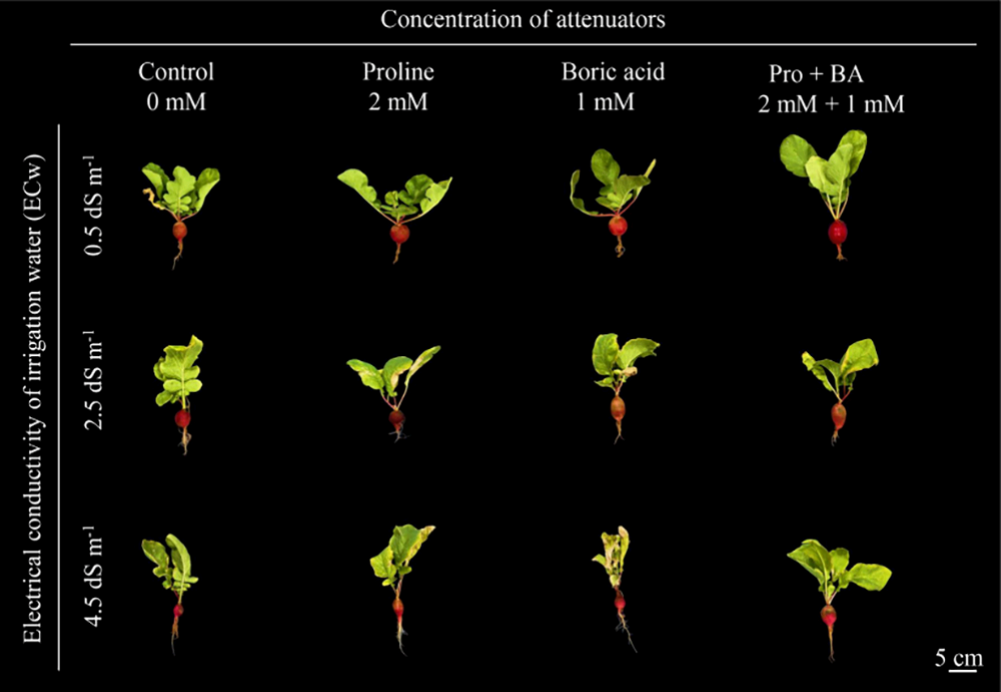
Radish plants under levels of electrical conductivity of irrigation water (ECw) and exogenous application of proline, boric acid, and proline + boric acid (Pro + BA).

The root diameter (RD) varied according to the salinity level and the type of attenuator used (Figures 2C and[Fig fig3]). Under 2.5 dS m^–1^, the application of proline promoted an increase by 15.04%, while boric acid promoted an increase of 13.20%. At the same level (2.5 dS m^–1^), the proline and boric acid combination also had a positive effect, with an increase of 10.02% (Figure 2C). At the 4.5 dS m^–1^ level, the combination of proline and boric acid provided a 7.84% increase over the control (Figure 2C).

The combined application of proline and boric acid (Pro + BA) provided increases in the physiological variables evaluated in the moderate (2.5 dS m^–1^) and severe (4.5 dS m^–1^) salinity levels (Figure 4). At a level of 2.5 dS m^–1^, the use of combined attenuators (Pro + BA) promoted increases of 19.27% in the net assimilation rate of CO_2_ (A), 10.16% in stomatal conductance (gs), and 16.77% in transpiration (E) (Figure 4A–C). In turn, the internal concentration of CO_2_ (Ci) was increased by 5.47% with the application of Pro + BA under a saline level of 4.5 dS m^–1^ (Figure 4D).

**Figure 4:**
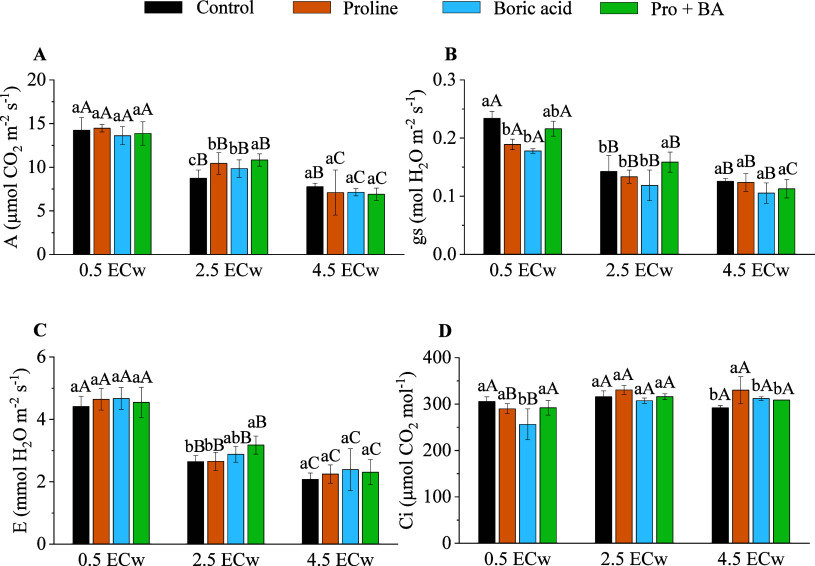
Net assimilation rate of CO_2_ (*A*) (A), stomatal conductance (gs) (B), transpiration (*E*) (C), and internal concentration of CO_2_ (Ci) (D) of radish plants under levels of electrical conductivity of irrigation water (ECw) and exogenous application of proline, boric acid, and proline + boric acid (Pro + BA). Means followed by the same lowercase letters do not differ for attenuations, and means followed by the same uppercase letters do not differ for ECw by Tukey’s test at 5% probability.

The application of attenuating agents resulted in increased levels of photosynthetic pigments (Figure 5). At a level of 2.5 dS m^–1^, the application of boric acid promoted increases of 19.88% in chlorophyll a, 19.91% in chlorophyll b, and 19.89% in total chlorophyll compared to the control (without attenuation) (Figure 5). Under 4.5 dS m^–1^, chlorophyll b showed increments of 15.25% with proline, 20.00% with boric acid, and 17.70% with the combination of proline + boric acid (Figure 5). At this same saline level, the total chlorophyll levels increased by 9.73% with the application of proline, 11.06% with boric acid, and 11.58% with the combination of proline and boric acid (Figure 5).

**Figure 5:**
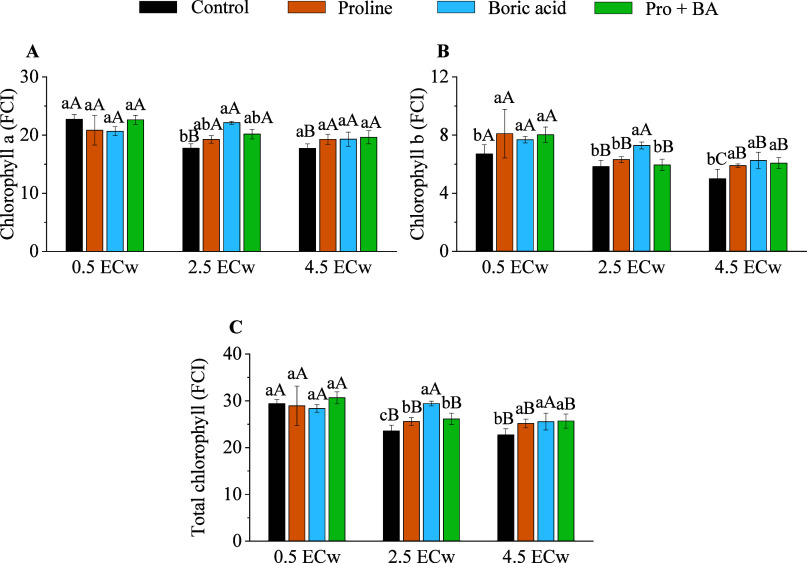
Chlorophyll a (A), chlorophyll b (B), and total chlorophyll (C) of radish plants under levels of electrical conductivity of irrigation water (ECw) and exogenous application of proline, boric acid, and proline + boric acid (Pro + BA). Means followed by the same lowercase letters do not differ for attenuations, and means followed by the same uppercase letters do not differ for ECw by Tukey’s test at 5% probability.

The application of the attenuators under salt stress conditions influenced the total soluble solids’ (TSS) and pulp firmness. At the same time, the pH of the applied attenuators was not influenced (Figure 6). At a level of 2.5 dS m^–1^, boric acid increased by 12.08%, while at a level of 4.5 dS m^–1^, there was an increase of 19.70% (Figure 6A). Only an isolated effect of salinity levels was observed for pH, with a level of 4.5 dS m^–1^ presenting the highest values (Figure 6B). At a saline level of 2.5 dS m^–1^, the application of boric acid increased the firmness of the pulp by 11.31% compared to the control (Figure 6C). At the most severe salinity level (4.5 dS m^–1^), the combination of proline and boric acid provided a 30.78% increase in pulp firmness (Figure 6C).

**Figure 6:**
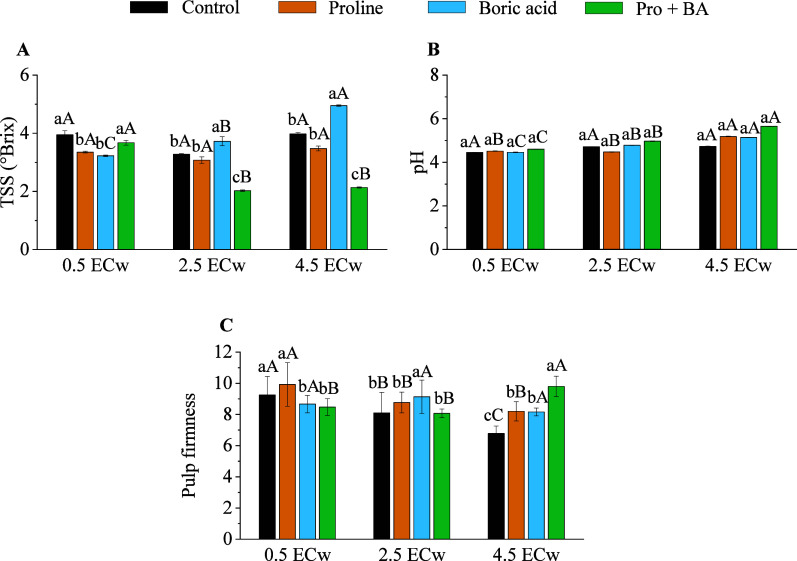
Total soluble solids (TSS) (A), pH (B), and pulp firmness (C) of radish plants under levels of electrical conductivity of irrigation water (ECw) and exogenous application of proline, boric acid, and proline + boric acid (Pro + BA). Means followed by the same lowercase letters do not differ for attenuations, and means followed by the same uppercase letters do not differ for ECw by Tukey’s test at 5% probability.

The application of attenuators significantly influenced the accumulation of phenolic compounds in plants under salt stress (Figure 7). At the moderate saline level (2.5 dS m^–1^), the treatment with boric acid promoted an increase of 26.32% in the flavonoid content and 26.91% in the anthocyanin content (Figure 7A and B). At a level of 4.5 dS m^–1^, the application of proline resulted in an even greater increase, reaching 45.05% for flavonoids and 55.18% for anthocyanins, compared to the treatment without the application of attenuators (Figure 7A and B).

**Figure 7:**
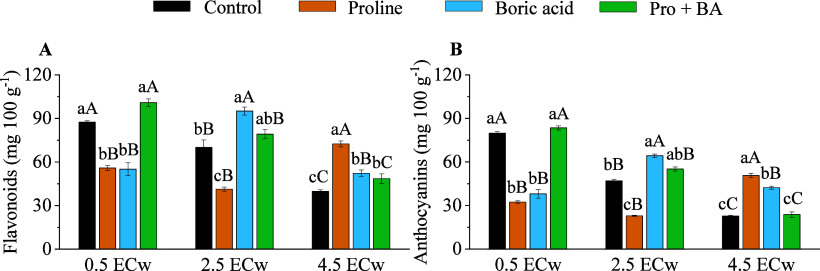
Flavonoids (A) and anthocyanins (B) in roots of radish plants under levels of electrical conductivity of irrigation water (ECw) and exogenous application of proline, boric acid, and proline + boric acid. Means followed by the same lowercase letters do not differ for attenuations, and means followed by the same uppercase letters do not differ for ECw by Tukey’s test at 5% probability.

Principal component analysis revealed that the first two components (PC1 and PC2) explain 74.02% of the total variability in the data, with PC1 (63.30%) primarily differentiating characteristics related to growth and quality, such as firmness, number of leaves, leaf area, and chlorophyll b. At the same time, pH and Ci are associated with negative values (Figure 8). PC2 (10.72%) highlighted biochemical compounds like flavonoids and anthocyanins. The treatments with irrigation water of higher electrical conductivity (4.5 dS m^–1^) showed greater dispersion, suggesting greater variability in the salinity responses. In comparison, those with a lower salinity (0.5 dS m^–1^) were more closely grouped. These results indicate that the effects of salinity and biostimulants have a significant influence on plants’ physiological responses.

**Figure 8:**
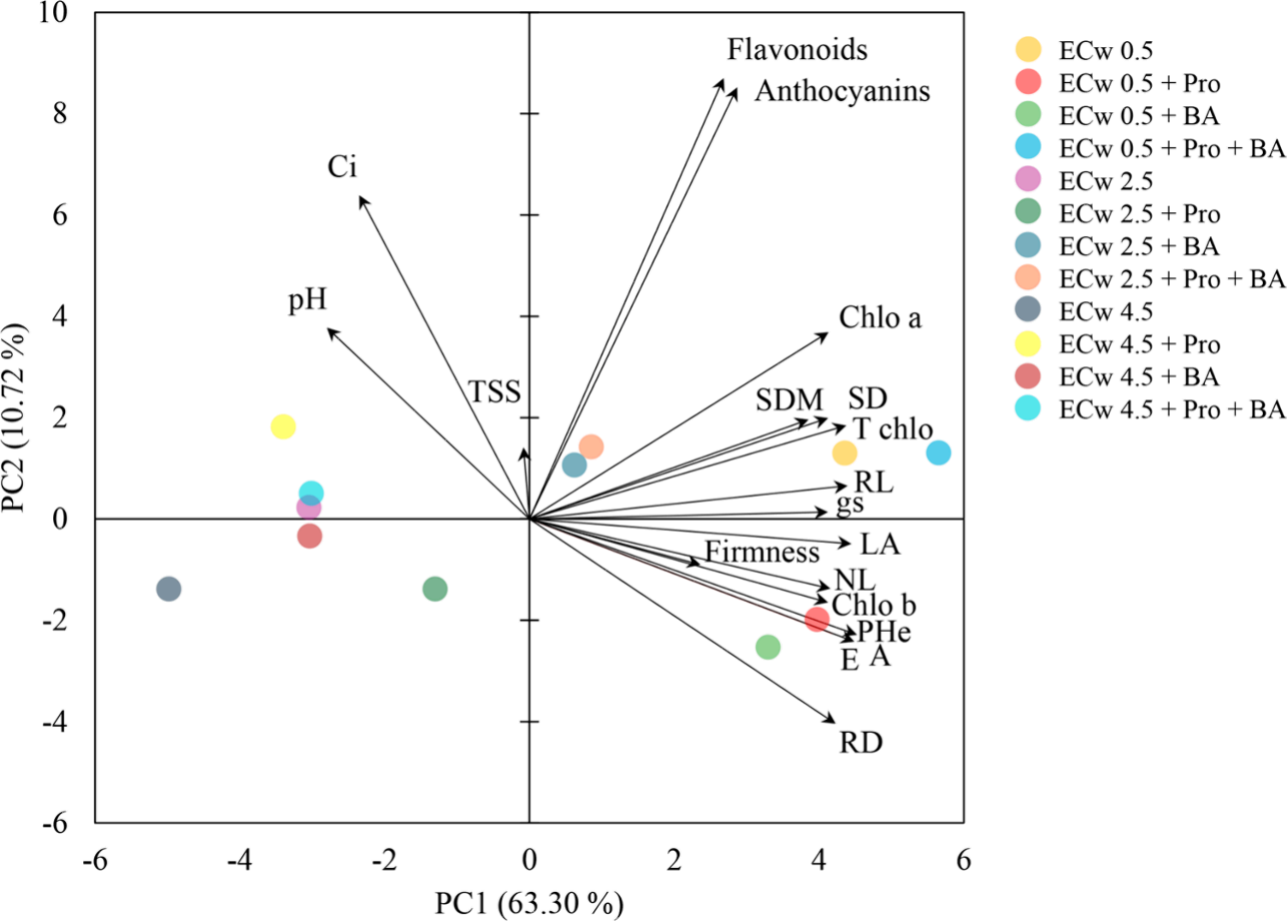
Principal component analysis between variables and treatments (levels of electrical conductivity of irrigation water and attenuating factors) in radish plants. ECw: electrical conductivity of irrigation water; Pro: proline; BA: boric acid; PHe: plant height; SD: stem diameter; NL: number of leaves; LA: leaf area; SDM: dry mass of the aerial part; RL: root length; RD: root diameter; A: net CO_2_ assimilation rate; gs: stomatal conductance; E: transpiration; Ci: internal concentration of CO_2_; Chlo a: chlorophyll a; Chlo b: chlorophyll b; T chlo: total chlorophyll; TSS: total soluble solids; pH: hydrogen potential.

The most significant positive correlations in Pearson’s analysis were observed among the variables: net CO_2_ assimilation with root diameter (0.93), leaf area, and root length (0.90); transpiration with root diameter (0.91), leaf area (0.87), root length, plant height, and number of leaves (0.85). In addition, a strong positive correlation was observed between chlorophyll b and plant height (0.94; Figure 9). These correlations suggest that physiological traits, including gas exchange and photosynthetic pigments, significantly influence plant growth and development (Figure 9). On the other hand, pH showed negative correlations with net CO_2_ assimilation rate (−0.77), root diameter (−0.77), stomatal conductance (−0.65), transpiration (−0.64), and root length (−0.63), suggesting a possible pH regulating effect on plant physiological processes (Figure 9). On the other hand, the internal concentration of CO_2_ showed a negative correlation with the root diameter (−0.62) (Figure 9). These results suggest that pH and internal CO_2_ concentration may play a relevant role in plant physiological processes, possibly affecting fruit quality (Figure 9).

**Figure 9:**
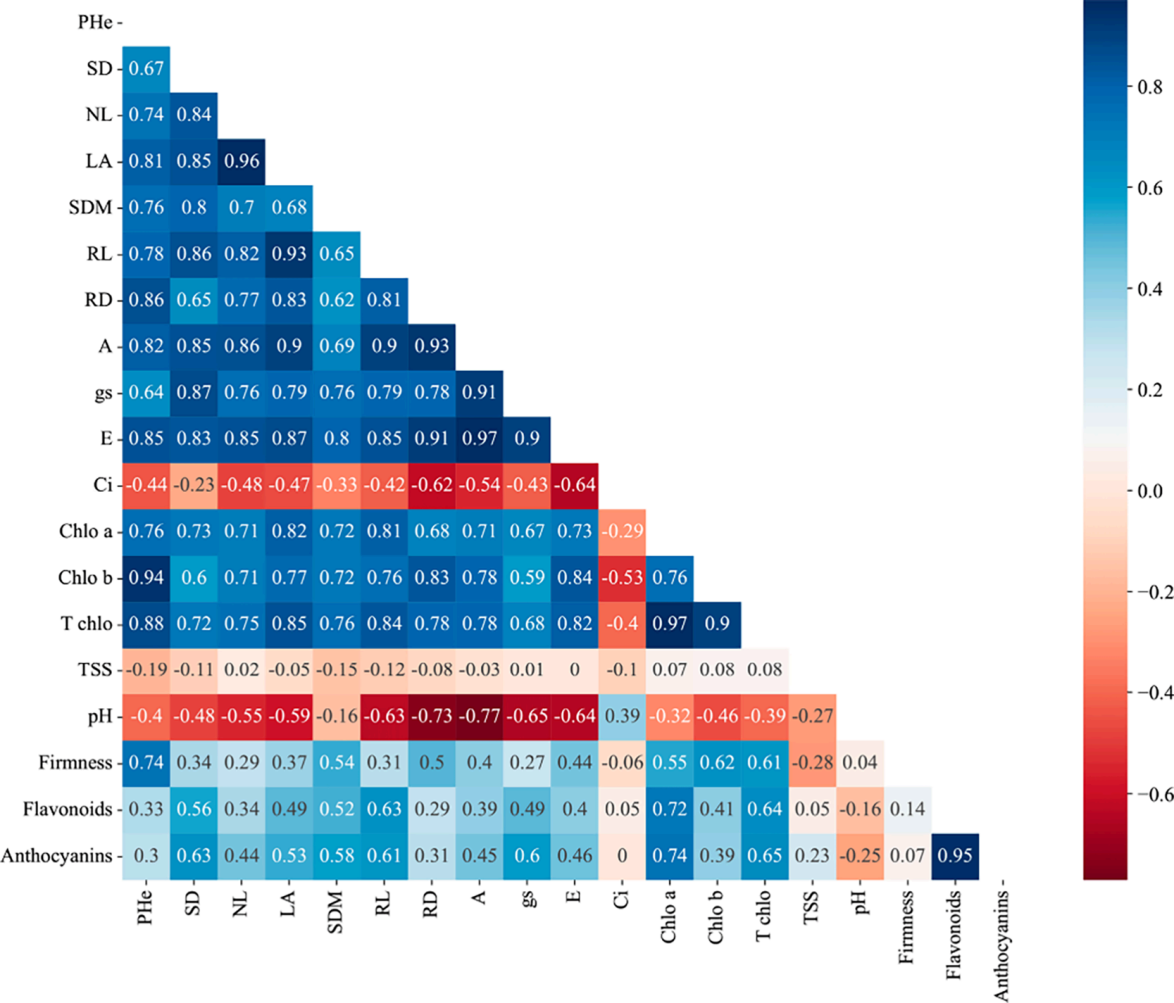
Pearson’s correlation analysis between the variables analyzed. ECw: electrical conductivity of irrigation water; Pro: proline; BA: boric acid; PHe: plant height; SD: stem diameter; NL: number of leaves; LA: leaf area; SDM: dry mass of the aerial part; RL: root length; RD: root diameter; A: net CO_2_ assimilation rate; gs: stomatal conductance; E: transpiration; Ci: internal concentration of CO_2_; Chlo a: chlorophyll a; Chlo b: chlorophyll b; T chlo: total chlorophyll; TSS: total soluble solids; pH: hydrogen potential.

## 3 Discussion

Plant height increased with the application of attenuators alone and in combination at moderate and severe saline levels, indicating that the maintenance of growth at height may be related to the greater availability of compounds essential for cell division and expansion, as well as to the regulation of phytohormones involved in cell elongation and differentiation.^37^ The stem diameter showed a greater response in an ECw of 2.5 dS m^–1^ for the combination of the compounds, while in an ECw of 4.5 dS m^–1^, proline and boric acid applied in isolation were more effective, indicating that each compound can act differently according to the intensity of stress, possibly modulating different physiological mechanisms.^38^ Proline may be involved in osmoprotection, regulating water balance, reducing oxidative damage, and stabilizing cellular structures.^22^ In addition, proline can play a role in regulating ROS, mitigating oxidative stress by activating antioxidant enzymes such as superoxide dismutase and catalase, which help protect plant cells from damage under saline conditions.^20^ Our results align with previous studies, demonstrating the beneficial effect of proline in mitigating salt stress in alfalfa,^32^ maize,^21^ and sorghum.^39^

Boric acid can influence cell wall integrity and lignin deposition, thereby contributing to stem strength and maintaining cell turgidity under salt stress.^40^ The influence of boric acid was also observed in the number of leaves, which followed the same trend, showing better performance in an ECw of 4.5 dS m^–1^ when applied alone, possibly due to its role in the biosynthesis of cell wall polysaccharides, thereby promoting greater structural stability and resistance to salt stress.^15^ Akden et al.,^28^ evaluating grapevines under salt stress, indicated that concentrations of 0.5 and 1 mM boric acid reduced the negative effects caused by salt stress. In comparison, a concentration of 2 mM had a negative effect, causing toxicity in the plants.

The leaf area was one of the most responsive parameters, showing significant increases with the combination of proline and boric acid at moderate and severe saline levels. The reduction of oxidative stress may have preserved the integrity of chloroplasts, favoring the stability of photosynthetic pigments and increasing the efficiency of electron transport in photosynthesis.^41^ Maintaining a larger leaf area allows for greater light interception and carbon fixation, increasing the metabolic activities of plants under saline conditions.^42^ Similarly, in the aerial part’s dry mass, the efficiency of attenuating agents was observed in reducing the negative effects of salt stress, indicating that proline and boric acid acted in combination to increase plant biomass.^43^

The variables related to the roots, such as the root length and diameter, responded differently to the application of the compounds. Boric acid favored root growth, promoting root elongation and thickening, while proline helped maintain metabolic activity, ensuring water and nutrient absorption even under salt stress.^16^ The effect of these combined compounds suggests that they act through complementary mechanisms, benefiting both the structure and function of the roots in saline environments.

In addition to the effects on growth, the combined application of proline and boric acid promoted improvements in the net CO_2_ assimilation rate, stomatal conductance, and transpiration, demonstrating the importance of these compounds in maintaining photosynthetic activity under salt stress. The increase in the internal concentration of CO_2_ in an ECw of 4.5 dS m^–1^ indicates that attenuators may be associated with mechanisms that regulate stomatal closure to minimize water losses while favoring carbon assimilation and improving water use efficiency.^44^

The greater effectiveness of the proline and boric acid combination at 2.5 dS m^–1^ may be due to moderate salinity still allowing for effective physiological adjustments, such as osmotic regulation, antioxidant activity, and membrane stability.^43^ At 4.5 dS m^–1^, the severity of salt stress may limit these responses, reducing nutrient uptake (e.g., boron) and overloading protective mechanisms, such as proline-mediated ROS detoxification, thereby decreasing the efficacy of the combined treatment.

The positive effects of attenuators were also observed in photosynthetic pigments. Applying boric acid alone resulted in greater increases in chlorophyll content under moderate salinity, indicating a possible role in stabilizing the chloroplast structure and protecting against oxidative degradation.^45^ At the highest salinity level (4.5 dS m^–1^), both proline and boric acid effectively maintained chlorophyll contents alone or in combination. The observed protective effect may be related to the treatment’s ability to preserve the integrity and functionality of the photosystems and regulate nitrogen metabolism, a key factor in the synthesis and maintenance of photosynthetic pigments, especially under salt stress conditions.^46^

Boric acid significantly affected total soluble solids and pulp firmness, especially under moderate and severe salt stress conditions. At moderate salinity levels (2.5 dS m^–1^), boric acid effectively increased the level of TSS, possibly due to its action in osmotic regulation and stabilization of plant cells. At higher salinity levels (4.5 dS m^–1^), boric acid was particularly effective in maintaining pulp firmness, suggesting that its application helped improve water retention and decrease loss of firmness under salt stress.^47,48^

The application of attenuators also positively influenced the accumulation of phenolic compounds, such as flavonoids and anthocyanins, which play a crucial role in protecting plants from oxidative stress damage.^49^ Proline, especially at the highest saline level (4.5 dS m^–1^), efficiently increases the number of these compounds. It suggests that, in addition to its osmotic effect, proline may activate antioxidant mechanisms that help reduce oxidative stress and protect the plant.^23,50^ Boric acid, in turn, demonstrated a greater efficacy at moderate salinity levels, promoting a significant increase in the content of flavonoids and anthocyanins. The effectiveness of boric acid at moderate salinity levels highlights its role in modulating the antioxidant response, thereby aiding in the maintenance of cellular function and metabolic stability in plants under salt stress conditions.^51^

Applying proline in combination with other compounds effectively mitigates the deleterious effects of salt stress. In wheat, its association with ascorbic acid attenuated the negative effects of salinity,^52^ while in rice, the combination with glycine betaine improved tolerance to salt stress.^53^ Similar results were observed in beans when proline was applied in combination with silicon.^54^ In addition, Salem^55^ found that applying boron associated with salicylic acid significantly increased the yield of millet plants grown in saline soil. Similarly, Younis et al.^56^ observed that combining boron and saponin improved the growth and physiological aspects of sweet potatoes under salinity conditions.

## 4 Conclusions

The application of the attenuators significantly influenced plant growth, physiological responses, and postharvest quality under saline conditions, with effects varying according to the salinity level and the specific variable analyzed. Proline and boric acid demonstrated the potential to mitigate the negative impacts of salinity, particularly when applied in combination, which was effective at moderate (2.5 dS m^1^) and severe (4.5 dS m^1^) salinity levels. The synergistic action of proline and boric acid appears to enhance osmotic regulation, preserve cellular integrity, and maintain essential physiological functions, such as photosynthesis and transpiration, under high-salinity conditions. From a practical perspective, these results provide valuable insights for agricultural practices, suggesting that utilizing these compounds may be an effective strategy for mitigating salt stress in radish crops. Therefore, the findings may assist producers in adopting these low-cost treatments as part of their management strategies to improve the yield and quality in large-scale radish production, particularly under saline conditions. However, given that the study was conducted in a greenhouse, further studies under field conditions are needed to validate the practical applicability of these treatments in agricultural systems. Future studies could investigate the long-term effects of these treatments under varying environmental conditions and in different crops, providing essential data for the combined use of these substances on a large scale.

## 5 Methods

### 5.1 Experimental Location and Climate Conditions

The experiment, conducted from September 26 to November 1, 2024, was carried out in a greenhouse belonging to the Department of Agronomic and Forestry Sciences of the Federal Rural University of the Semiarid (UFERSA), located in the municipality of Mossoró, Rio Grande do Norte, Brazil (5°12′28″ S, 37°19′04′′ W, altitude of 24 m). The region has a hot and dry climate, classified as BSh according to the Köppen climate classification, with a predominantly dry season and rainfall concentrated in the summer.^57^ During the experimental period, the temperature and relative humidity of the air were monitored daily by using a digital thermohygrometer installed on site, with the resulting values shown in Figure 10.

**Figure 10:**
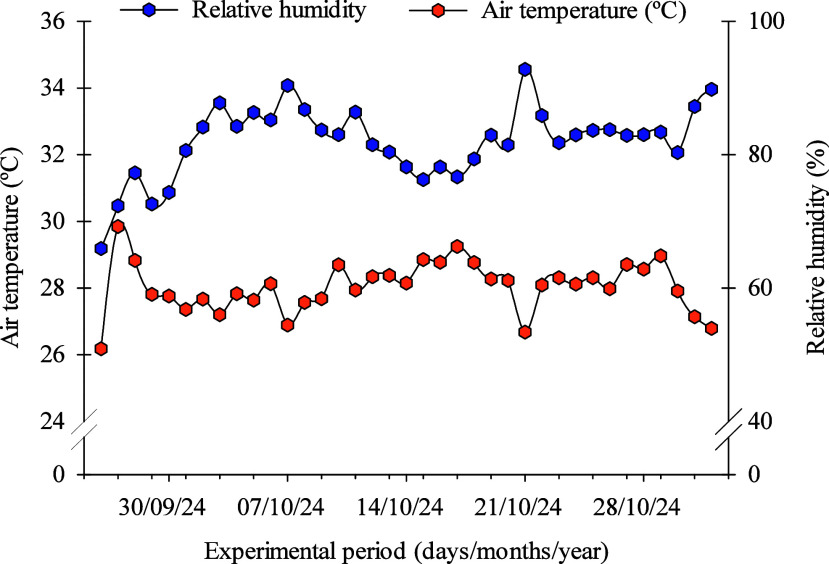
Air temperature and relative humidity during the experimental period.

### 5.2 Experimental Design and Treatments

The experimental design employed randomized blocks, utilizing a 3 × 4 factorial scheme with four replications, resulting in a total of 48 experimental units and one plant per experimental plot. The treatments included three levels of electrical conductivity of the irrigation water [ECw 1: 0.5 dS m^–1^ (without stress); ECw 2: 2.5 dS m^–1^ (moderate stress); and ECw 3: 4.5 dS m^–1^ (severe stress)][Bibr ref58] and four attenuating treatments: control (no application), proline (5 mM),^59^ boric acid (1 mM),^28^ and the combination of proline and boric acid (5 mM + 1 mM). The salt levels were prepared with sodium chloride (NaCl), using 1.17 g L^–1^ NaCl used to prepare the 2.5 dS m^–1^ solution and 2.34 g L^–1^ NaCl used for the 4.5 dS m^–1^ solution, both of which were stored in 60 L plastic containers. The electrical conductivity of water was monitored every 3 days using a portable digital conductivity meter (Instrutherm, model CD-880). The chemical attributes of the salt levels used are presented in Table 1.

**Table 1:** Chemical Attributes of the Salt Levels Used in the Experiment^a^

parameter (unit)	ECw 1	ECw 2	ECw 3
pH (water)	8.8	8.9	8.7
electrical conductivity (dS m^–1^)	0.5	2.50	4.50
potassium (mmol_c_ L^–1^)	0.25	0.24	0.24
sodium (mmol_c_ L^–1^)	4.23	31.41	51.49
calcium (mmol_c_ L^–1^)	0.7	0.8	0.9
magnesium (mmol_c_ L^–1^)	1.9	1.3	1.3
chloride (mmol_c_ L^–1^)	3	25.6	48.6
carbonate (mmol_c_ L^–1^)	0.6	0.8	0.4
bicarbonate (mmol_c_ L^–1^)	2.8	2.9	2.7
sodium adsorption ratio (mmol L^–1^)^0^ ^.5^	3.7	30.7	49.1
hardness (mg L^–1^)	130	105	110
cations (mmol_c_ L^–1^)	7.08	33.75	53.93
anions (mmol_c_ L^–1^)	6.4	29.3	51.7

a ECw: Electrical conductivity of irrigation water.

### 5.3 Plant Material, Soil, and Irrigation

The seeds of the radish variety “Crimson Gigante” were sown directly in polyethylene pots with a volumetric capacity of 3.0 dm^3^ using the direct seeding method. The pots were filled with soil collected from an area near the research site. Soil samples were sent to the Soil, Water, and Plant Laboratory of UFERSA to analyze their physicochemical attributes according to the procedures described in the Embrapa protocol.^60^ The physicochemical characteristics of the soil are listed in Table 2. The soil presented low electrical conductivity (0.05 dS m^–1^) and a low exchangeable sodium percentage (2%), indicating nonsaline and nonsodic conditions (Table 2). This low baseline salinity allowed for an accurate assessment of the effects of NaCl added through irrigation water, thereby minimizing background interference from soil salinity.

**Table 2:** Physicochemical Attributes of the Soil Used in the Experiment

physical properties of the soil		
parameter	unit	value
coarse sand	g kg^–1^	480
fine sand	g kg^–1^	330
silt	g kg^–1^	140
clay	g kg^–1^	50
textural class		sandy loam

chemical properties of the soil		
parameter	unit	Value
pH	water 1:2.5	6.90
electrical conductivity	dS m^–1^	0.05
potassium	mg dm^–3^	109.8
sodium	mg dm^–3^	20.8
phosphorus	mg dm^–3^	75.3
calcium	cmol_c_ dm^–3^	3.54
magnesium	cmol_c_ dm^–3^	0.66
aluminum	cmol_c_ dm^–3^	0
H^+^ + Al^3+^	cmol_c_ dm^–3^	0
sum of exchangeable bases	cmol_c_ dm^–3^	4.57
effective cation exchange capacity	cmol_c_ dm^–3^	4.57
total cation exchange capacity	cmol_c_ dm^–3^	4.57
base saturation	%	100
aluminum saturation	%	0
exchangeable sodium percentage	%	2

Daily irrigation was conducted using the weighing lysimetry method, in which the volume of evaporated or transpired water over a 24 h interval was determined. This procedure allowed for the necessary water replacement to maintain soil moisture at 80% of field capacity.^61^

### 5.4 Application of Treatments

The application of saline irrigation treatments commenced 14 days after sowing (DAS), corresponding to the early vegetative stage of the radish plants, and was maintained daily until the end of the experiment (34 DAS), encompassing the entire growth cycle, including both vegetative and reproductive stages. At 14 DAS, the attenuating treatments, consisting of proline and boric acid, were applied via foliar spray and reapplied weekly, totaling three applications until harvest. The solutions were prepared with deionized water and 0.05% Tween 80, a nonionic surfactant used to improve the adhesion and penetration of the substances through the leaf cuticle. Spraying was performed in the late afternoon by using a manual spray bottle, ensuring full coverage of the adaxial and abaxial leaf surfaces. Control plants, which did not receive the attenuating compounds, were sprayed with deionized water containing only the surfactant.

### 5.5 Variables Analyzed

At 34 DAS, plant height (PHe) was measured using a millimeter ruler; stem diameter (SD) was measured with a digital caliper, and the number of leaves per plant (NL) was counted directly. Additionally, productivity variables, including diameter (RD) and root length (RL), were measured by using a digital caliper. The leaf area (LA, cm^2^) was determined by multiplying the length and width of the leaves (LW), according to the equation proposed by Aminifard et al.:^62^ LA = 0.847 (LW) + 29.39.

After the measurements, the plants were separated into shoots and roots. The shoot was dried in an oven with forced air circulation at 65 °C for 72 h until it reached a constant mass for subsequent quantification of the shoot dry mass (MDS). Then, the samples were weighed on a semianalytical scale with a precision of 0.001 g, and the results were expressed in grams per plant.

Gas exchange analyses were performed to measure the net assimilation rate of CO_2_ (A), stomatal conductance (gs), transpiration (E), and internal concentration of CO_2_ (Ci). Measurements were taken between 8 and 10 a.m. on completely expanded leaves with no signs of damage located in the middle third of the plants. A portable infrared gas analyzer (IRGA) (model GFS-3000, Walz) was used, configured with a photosynthetically active photon density of 1200 μmol m^–2^ s^–1^, air flow of 750 μmol s^–1^, atmospheric CO_2_ at 400 μmol mol^–1^, relative humidity of 60%, and temperature of 25 °C.

The chlorophyll a, chlorophyll b, and total chlorophyll concentrations were evaluated with a portable chlorophyll meter (ClorofiLog, model CFL1030, Falker). The measurements were carried out on two leaves per plant, located in the middle third of the plant, and the results were expressed in the Falker Chlorophyll Index (FCI).

Fresh roots were used to quantify the physicochemical variables. Total soluble solids (°Brix) were measured using a refractometer, pulp firmness was evaluated with a manual penetrometer, and flavonoids and anthocyanins were quantified by spectrophotometry, as described by Francis and Markakis.^63^ The pH was determined with a pH meter.

### 5.6 Data Analysis

The data were submitted for analysis of variance (F test, *p* ≤ 0.05) after verifying the homogeneity of variances using the Levene test and the normality of the residuals using the Shapiro–Wilk test. In cases of significance, the means were compared using Tukey’s test (*p* ≤ 0.05). The analyses were performed using R statistical software.^64^
